# A Computational Evaluation of Minimum Feature Size in Projection Two-Photon Lithography for Rapid Sub-100 nm Additive Manufacturing

**DOI:** 10.3390/mi15010158

**Published:** 2024-01-21

**Authors:** Rushil Pingali, Harnjoo Kim, Sourabh K. Saha

**Affiliations:** G.W. Woodruff School of Mechanical Engineering, Georgia Institute of Technology, Atlanta, GA 30332, USA; rushilpingali@u.northwestern.edu (R.P.); harnjookim@gatech.edu (H.K.)

**Keywords:** 3D printing, multi-photon polymerization, femtosecond laser processing, photopolymerization, finite element modeling, dynamic programming, high-performance computing

## Abstract

Two-photon lithography (TPL) is a laser-based additive manufacturing technique that enables the printing of arbitrarily complex cm-scale polymeric 3D structures with sub-micron features. Although various approaches have been investigated to enable the printing of fine features in TPL, it is still challenging to achieve rapid sub-100 nm 3D printing. A key limitation is that the physical phenomena that govern the theoretical and practical limits of the minimum feature size are not well known. Here, we investigate these limits in the projection TPL (P-PTL) process, which is a high-throughput variant of TPL, wherein entire 2D layers are printed at once. We quantify the effects of the projected feature size, optical power, exposure time, and photoinitiator concentration on the printed feature size through finite element modeling of photopolymerization. Simulations are performed rapidly over a vast parameter set exceeding 10,000 combinations through a dynamic programming scheme, which is implemented on high-performance computing resources. We demonstrate that there is no physics-based limit to the minimum feature sizes achievable with a precise and well-calibrated P-TPL system, despite the discrete nature of illumination. However, the practically achievable minimum feature size is limited by the increased sensitivity of the degree of polymer conversion to the processing parameters in the sub-100 nm regime. The insights generated here can serve as a roadmap towards fast, precise, and predictable sub-100 nm 3D printing.

## 1. Introduction

Two-photon lithography (TPL) is a state-of-the-art nanoscale additive manufacturing technique that is capable of fabricating truly three-dimensional (3D) structures with feature sizes on the scale of 100 nm [1,2,3]. It is a direct laser writing (DLW) process that is capable of precise and digitally controlled polymer printing. TPL exploits the nonlinear nature of the two-photon absorption (TPA) process to print features smaller than the diffraction limit of light [4]. It utilizes focused beams of femtosecond laser to achieve high light intensities on the order of 1 TW/cm^2^ to achieve nonlinear light absorption in photocurable resins. In recent years, TPL has become the premier choice for manufacturing challenging 3D micro- and nano-scale structures with applications in fields as diverse as medicine [5,6], micro-robotics [7,8], anti-counterfeiting [7], high-energy-density physics [9], optics [10,11], photonics [12,13], energy storage [14], and engineered structural materials [15,16,17]. Numerous other applications related to semiconductors and quantum manufacturing can be enabled with further improvements in minimum feature size. However, it is challenging to rapidly print 3D structures with features that are 10s of nm in size using TPL. To overcome this challenge, we present a computational investigation of the limits of the minimum feature size achievable with the projection two-photon lithography (P-TPL) technique, which is a high-throughput variant of TPL. 

Traditional TPL relies on a point-by-point laser scanning scheme that produces a single volumetric pixel (i.e., voxel) at a time, and generates the 3D structure by overlapping the individual voxels [18]. This serial writing process has the disadvantage of being slow due to low linear scanning speeds between 10 µm s^−1^ and 100 mm s^−1^ [19,20]. Thus, there has been significant interest in increasing the throughput of TPL in order to foster wider adoption of this technology. Approaches for increasing the throughput include increasing the scanning speed of serial writing, parallelizing the writing, and combining the previous two approaches [21,22,23,24]. It is noteworthy that recent studies have demonstrated high scanning speeds up to and exceeding 1 m s^−1^ with serial TPL [25,26,27,28,29]. However, the printing of 3D structures with sub-100 nm feature sizes has not yet been demonstrated at these high speeds. Thus, achieving rapid sub-100 nm 3D printing remains a major challenge. Toward this goal, the serial writing approach is fundamentally limited by the unfavorable feature size versus throughput tradeoff. This is because the distance over which scanning must be performed to fill a 2D layer scales inversely with the feature size. Thus, the time required to print a layer increases with a decrease in the feature size. In contrast, the time required to print a 2D layer in P-TPL does not increase with a decrease in the feature size [21]. P-TPL replaces the serial writing scheme with a layer-by-layer parallel writing scheme that is enabled through the simultaneous spatiotemporal focusing of femtosecond light [21]. We have previously demonstrated that this parallelization increases the rate of printing by two to three orders of magnitude while printing sub-200 nm features [21]. P-TPL demonstrates favorable scaling of the feature size versus throughput, because the area within each layer can be filled at once, irrespective of whether it is filled with small or large voxels. Thus, improving the minimum feature size in P-TPL is a promising approach for achieving rapid sub-100 nm 3D printing. 

It is challenging to improve the minimum feature size in P-TPL due to the lack of processing knowledge about the process limits. It is well known within the TPL literature that smaller voxels can be generated at lower light dosages [30]. However, the size of the voxel cannot be reduced to infinitesimally small values by progressively reducing the dosage because of the thresholding behavior. It has been observed that writing in serial TPL abruptly stops if the dosage falls below a threshold value [31,32]. The feature size corresponding to this threshold dosage represents the minimum printable feature size. Although past studies have demonstrated that this limit can be tuned by tuning the properties of the photopolymer resin [33,34], quantitative models that can explain or predict it are not available. Consequently, it is challenging to rationally determine how one may tune the various material properties and processing conditions to optimize the minimum feature size. This challenge is further compounded in P-TPL because it has a larger design space, which arises from its ability to tune the light dosage through image projection [35]. To overcome these challenges, we present a physics-based computational model of photopolymerization and interrogate it to characterize the minimum feature size limit of P-TPL as a function of the processing conditions and properties of the photopolymer resin. 

Our computational model was developed by implementing a finite elements (FE)-based reaction–diffusion model of photopolymerization in P-TPL. It was based on our previous work on the modeling of P-TPL, which demonstrated the ability to predict feature sizes with reasonable accuracy [36]. That work advanced previous attempts at modeling TPL [32,37,38] by tracking the effect of individual laser pulses, modeling the diffusion of chemical species in the photoresist, and using a solubility threshold to predict the feature sizes. The model was specifically tuned for the unique aspects of P-TPL, which involves different length and time scales than serial TPL. Specifically, P-TPL projects 2D images over areas larger than 100 μm × 100 μm at once, unlike the point-by-point printing of serial TPL. Additionally, projection within each plane occurs through a series of discrete illumination events, because the repetition rate of the amplifier-based femtosecond laser that is used in P-TPL is on the order of 1 kHz [21,35]. This repetition rate is significantly smaller than the 10s of MHz repetition rates of oscillator-based femtosecond lasers that are prevalent in fast-scanning serial TPL. Thus, in contrast to the near-continuous exposure in serial TPL, each femtosecond pulse in P-TPL is followed by a long dark period, which leads to a fundamentally different time scale of exposure. By accounting for these complexities, the reaction–diffusion model is capable of predicting printing outcomes in a fraction of the time that would otherwise be required to set up and run a physical experiment [36]. 

Although FE models open up the possibility to advance our understanding of the capabilities and limitations of P-TPL, it is challenging to computationally characterize the process performance over a large set of parameters. This is because it takes several minutes to solve each FE model. Here, we overcame this challenge by implementing a dynamic programming scheme to reduce the time complexity of the computations. Dynamic programming schemes enable reducing the computation time by breaking a problem down into smaller parts and reusing the results of those parts in multiple solutions [39]. Our dynamic programming scheme was implemented on high-performance computing (HPC) infrastructure to further reduce the computation time by taking advantage of parallel computing. This enabled us to perform a full-factorial characterization of the effect of the process parameters over a set of more than 10,000 parameter combinations of optical power, exposure time, projected linewidth, and the concentration of photoinitiator in the resin. These studies help us to understand whether P-TPL is fundamentally capable of achieving sub-100 nm or even sub-50 nm feature sizes. Our studies demonstrate that there is no physics-based limit to the feature sizes, but achieving sub-100 nm printing is practically challenging due to the enhanced sensitivity of printing to the process parameters.

## 2. Materials and Methods

### 2.1. P-TPL Process

**Figure 1:**
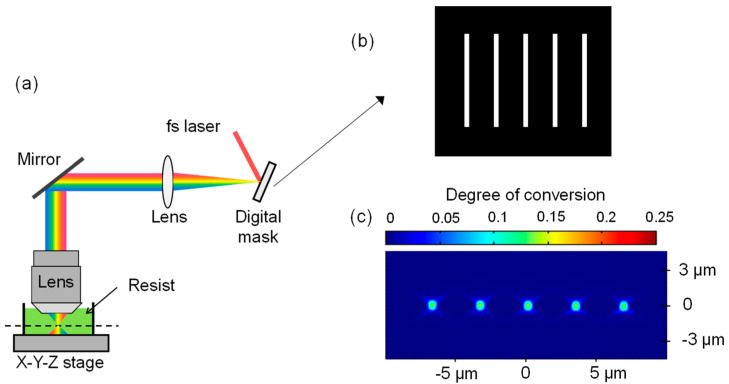
(**a**) Schematic of P-TPL technique, (**b**) representative bitmap image that was projected on the digital mask, and (**c**) representative simulated degree of conversion that results from projecting the mask. Figures have been adapted from our past work [35], which was published under a CC BY license.

A schematic of the P-TPL process that was modeled here is shown in Figure 1. Femtosecond (fs) laser light is diverted off a digital micromirror device (DMD), which acts as a digital mask to control which pixels are illuminated for printing. This allows for the projection and printing of an entire 2D layer at once, following which, the mask can be easily updated to the next image. Temporal focusing is implemented to ensure that the required light intensity for printing is achieved only in the focal plane, where the duration of the laser pulse is shortest and the intensity is highest. This limits printing to the focal plane and preserves the depth resolvability. Temporal focusing refers to the process of broadening the duration of each fs pulse in regions away from the focal plane and compressing the pulse back to its shortest duration at the focal plane [40]. Since an entire 2D layer is printed at once in this manner, the printing throughput is insensitive to the geometry being printed in each layer and the proportion of the layer that is solid. Furthermore, arbitrary patterns can be printed in each layer with ease, since *X*-*Y* stage movement is not necessary. The stage is moved in the *Z* direction to position the focal plane as desired to print 3D structures in a layer-by-layer fashion.

### 2.2. Finite Elements Model of P-TPL

Our FE model is based on our previous works [35,36], and it is briefly summarized here. Photopolymerization in P-TPL is driven by pulses of femtosecond light that are generated at a low repetition rate, which is 5 kHz for our modeled system. Upon the absorption of each pulse of light, the photoinitiator molecules in the resin generate radicals, i.e., reactive species. Here, we consider the radical generation from each pulse to be an instantaneous event because the duration of each pulse is on the order of 100 fs and subsequent pulses arrive after gaps of 200 μs. Each absorption event transforms a certain proportion of available photoinitiator (PI) molecules into primary radicals (R*). This proportion can be quantified from the amount of light absorbed via two-photon absorption and the properties of the photoinitiator [35]. This relationship is shown in Equation (1). Here, *σ*^(2)^ is the two-photon cross-section of the photoinitiator, Φ is the quantum yield of the radical formation reaction, *h* is the Planck’s constant, and *ν* is the frequency of light. The square bracket notation is used here to represent the concentration of the chemical species that is listed inside the brackets.

(1) $$∆\left[R^{*}\right]=\frac{{D_{p}\sigma }^{\left(2\right)}\Phi }{h^{2}v^{2}}[PI]$$

The prior version of the model used the squared intensity of the light field multiplied by the average duration of the light pulse as its optical input [36]. This has since been updated to use the optical dosage per pulse (*D*_p_), which accurately captures the spatially-varying pulse duration instead of simply averaging it over space [35]. *D*_p_ was evaluated by integrating the square of the instantaneous intensity with respect to time over the duration of the pulse. The optics simulation for the dosage evaluation is discussed in detail in the supporting materials of our past work [21]. The updated model also accounts for the decrease in the amount of photoinitiator available for further reaction due to the consumption after each pulse event. The quantum yield of the photoinitiation reaction was empirically calibrated, while the values of other parameters were obtained from the literature, as detailed in our past work [35]. These model parameters are listed in Table 1. The modeled photoresist is an acrylate-based photoresist that undergoes cross-linking through free radical polymerization. We simplify the optical model by considering that the photoresist exhibits minimal spherical aberrations. To ensure this, we consider that the photoresist has a refractive index of 1.52, which matches the index of the immersion oil of the objective lens. We believe that this is a reasonable simplification, because various functional photoresists that satisfy this index-matching criterion can be synthesized [41,42]. For the monomer component of the photoresist, we use the properties of pentaerythritol triacrylate, which is a popular monomer for TPL [32,43]. For the photoinitiator, we use the properties of a custom photoinitiator, which was used in our previous study [21].

Aside from radical generation, the chemistry of P-TPL progresses at microsecond to millisecond timescales and can be modeled accurately through a system of partial differential equations (PDEs), given here in Equations (2)–(7). Each of these reaction–diffusion equations relates the rate of change in the concentration of the chemical species on the left-hand side of the equation to the concentrations of the species on the right-hand side. Processing occurs via the formation of new covalent bonds between the monomer molecules of the resin (i.e., the photoresist), which leads to an increase in the degree of polymer conversion (*DOC*) via cross-linking. The primary radicals react with the monomer molecules (M) to generate secondary radicals (P*). These secondary radicals continue to react with the monomers to form cross-links and are regenerated during the cross-linking reaction. The primary and secondary radicals become inactive upon reacting with the dissolved oxygen (O_2_) in the resin. The cross-linking process stops when no free radicals remain in the resin. The *DOC* represents the fraction of the total monomer molecules that react to form the polymer. The parameters *k_p_*, *k_q_*, and *k_t_* represent the reaction rate constants for the polymerization, quenching, and the termination reactions, respectively. Equations (2) and (5) have spatial terms that represent the diffusion of those species (R* and O_2_) from regions of high concentration to regions of low concentration. The diffusion of the larger molecules is not modeled here because the diffusion of such molecules is expected to be significantly slower. The values of the reaction rate constants and diffusion coefficients are provided in Table 1. The polymerization rate coefficient *k*_p_ is modeled to decrease with an increase in the *DOC* (and thus the viscosity of the resin). The *k_p_* versus *DOC* relationship modeled here is based on values from the literature [32], and the value listed in Table 1 corresponds to the value at *DOC* = 0. For the range of values of *DOC* observed in this study, *k_p_* is obtained by linearly decreasing it by 6% of its initial value for every 1% increase in the *DOC*.

(2) $$\frac{d}{dt}\left[R^{*}\right]=-k_{p}\left[M\right]\left[R^{*}\right]-k_{q}\left[O_{2}\right]\left[R^{*}\right]+D_{R^{*}}\left(\frac{\partial^{2}\left[R^{*}\right]}{\partial x^{2}}+\frac{\partial^{2}\left[R^{*}\right]}{\partial z^{2}}\right)$$

(3) $$\frac{d}{dt}\left[M\right]=-k_{p}\left[M\right]\left[R^{*}\right]-k_{p}\left[M\right]\left[P^{*}\right]$$

(4) $$\frac{d}{dt}\left[P^{*}\right]=k_{p}\left[M\right]\left[R^{*}\right]-k_{t}\left[O_{2}\right]\left[P^{*}\right]$$

(5) $$\frac{d}{dt}\left[O_{2}\right]=-k_{q}\left[O_{2}\right]\left[R^{*}\right]-k_{t}\left[O_{2}\right]\left[P^{*}\right]+D_{O2}\left(\frac{\partial^{2}\left[O_{2}\right]}{\partial x^{2}}+\frac{\partial^{2}\left[O_{2}\right]}{\partial z^{2}}\right)$$

(6) $$\frac{d}{dt}\left[R^{x}\right]=k_{q}\left[O_{2}\right]\left[R^{*}\right]$$

(7) $$\frac{d}{dt}\left[P^{x}\right]=k_{t}\left[O_{2}\right]\left[P^{*}\right]$$

Equations (2)–(7) are solved using the FE method implemented in a COMSOL Multiphysics model similar to that described in our previous work [35]. By solving the PDE system over the specified geometry in a transient manner, the concentrations of each species at each point in space and time are calculated. The initial concentration of the monomer is set at 4 mol dm^−3^ and the initial concentration of the dissolved oxygen is set at 0.006 mol dm^−3^ [35]. The initial concentration of the PI varies over a range, and the initial concentration of all other species is set to 0 [35]. Regions with a *DOC* above a threshold value (*DOC_th_*) are considered to be printed. This threshold represents the *DOC* above which the cured polymer becomes insoluble in a solvent. The uncured sections of the resin can be dissolved away to only leave behind the cured, solidified polymer material. The value of *DOC_th_* is determined experimentally through Raman spectroscopy to be 6.8% [35]. It is noteworthy that, here, we only consider the solubility threshold for printing, without any consideration of the mechanical stability of the printed features, because the mechanical properties can be further improved after printing through photochemical curing [35]. Thus, applying the solubility threshold for printability provides a more accurate estimate of the minimum printable feature size.

### 2.3. Full-Factorial Design of Computational Experiments

While a naive approach to printing the smallest possible feature might be to simply reduce the optical power and size of the features in the projected image as much as possible, we hypothesize that this is not necessarily the optimal approach due to the underlying nonlinearity of the processing. TPL is a fundamentally nonlinear process through several mechanisms. First, two-photon absorption is nonlinear with respect to light intensity, unlike single-photon absorption [45]. Second, the free radicals produced upon two-photon absorption are rapidly quenched by oxygen or other inhibitors dissolved in the resin, leading to the reaction being suppressed entirely at low light intensities [46]. Third, the solubility of the cured polymer depends on the *DOC* so that regions of the resin with a low *DOC* remain soluble in the developer and can be washed away. Only regions with a *DOC* greater than a certain threshold (*DOC_th_*) are insoluble and preserved [4]. As a result of these nonlinearities, the printed features can be much smaller than the features in the projected image, and a combination of high power, low exposure, and large projected features may produce fine features.

A key factor that makes it more challenging to intuitively determine the optimal processing conditions that lead to the smallest feature size is the discrete nature of the pulsed projection in P-TPL. While each exposure event in P-TPL is only femtoseconds long, there are dark periods of 0.2 ms or greater between successive pulses due to the low repetition rate of the amplifier-based lasers [35]. This dark period is longer than the lifetime of the primary radicals, which is expected to be less than 10 μs [47]. In contrast, the dark period between the pulses in fast-scanning serial TPL systems, that are based on oscillator-based lasers, is on the order of 10 ns [48]. Thus, the duration of exposure in such serial TPL can be considered to be a continuous parameter, whereas exposure in P-TPL is a discrete parameter. This means that the exposure in P-TPL cannot be tuned with arbitrary precision to deliver precisely the light dosage required to achieve the *DOC_th_* and nothing more. As a result, it is possible that using a larger projected feature size or a higher power, for instance, could counterintuitively result in a smaller print, simply because the required number of pulses to print is fewer. Thus, the problem of determining the minimum feature size limits of P-TPL is non-trivial. Here, we characterize the process under different printing conditions and with different projections to clarify these limits.

In this study, we varied the following processing conditions: (i) the linewidth in the projected image, (ii) the average optical power of the projection beam, (iii) the initial concentration of the photoinitiator in the resin, and (iv) the number of laser pulses (i.e., the duration of exposure as a discrete variable). The projected images that were displayed on the DMD were standardized, and a representative image is shown in Figure 1b. The projected image comprised a set of five lines of the same width, spaced regularly at a period of 30 pixels (px). In our modeled system, each pixel width on the DMD was demagnified to 113 nm in the projected image. Due to the symmetry of these lines along their length, the simulation problem could be reduced to a 2D simulation in the *x*-*z* plane, which is computationally less demanding than the full 3D simulation. The *x*-direction lies along the width of the lines and the z-direction lies along the height of the lines. The ranges over which the parameters varied are listed in Table 2. With these four independent variables, a full-factorial experimental design resulted in 11,760 parameter combinations to be tested. For each combination of these parameters, the simulated width and height of the five printed lines were measured from the simulated *DOC* profile. It was observed that the central line was slightly larger than the other four lines due to optical effects. Therefore, to maintain consistency, we used the measurements of the central line to study the effects of the various process parameters. To rapidly and efficiently solve this large number of simulations, dynamic programming and high-performance computing (HPC) were leveraged.

### 2.4. Dynamic Prgramming Approach

In our FE simulations, we observed that the computation time is dominated by the duration over which exposure occurs, instead of the total duration of the simulated time span. For example, in a typical simulation experiment with a 2 ms exposure period followed by a 100 ms dark period, the numerical solver spends more than 60% of its computation effort on the exposure period. This is because the solver uses an adaptive time-stepping algorithm that slows it down near laser pulses, where there are rapid changes in species concentrations. Outside this regime, it is allowed to take large time steps in the dark period. This results in the time complexity of a P-TPL simulation being O(*n*), where *n* is the number of pulses.

As shown in Table 2, the independent variable with the greatest number of levels is exposure, with 49 possible values. However, simulations of a particular projected linewidth at a given power and [PI]_0_ for exposures of *n* pulses and *n* + 1 pulses both proceed exactly the same for the duration of the first *n* pulses. Since this is where most of the computation effort is required, it means that many calculations would be repeated over the course of the parametric sweep. To avoid this inefficiency, a dynamic programming approach involving memoization is implemented. Since the overall problem of the large parametric sweep has overlapping subproblems, partial solutions can be stored and reused instead of wastefully recalculating them [39]. A common dynamic programming implementation involves recursion, where partial solutions are recursively computed or reused if they already exist [39]. However, it is found that implementing such a recursive scheme for FE simulations is impractically memory intensive. Thus, a bottom-up approach based on iteration is implemented, whereby solutions for each combination of linewidth, power, and [PI]_0_ are computed sequentially in an increasing order of exposure time. After each pulse, the partial solution is saved, and it is reused in solving the models with higher exposures. This ensures that no calculations are repeated, and each simulation must compute the effect of only one more pulse from a preexisting solution. Thus, the time complexity is reduced to O(1), providing significant speedup.

While this dynamic programming approach results in performance gains for sweeps over different exposure values, it cannot reduce the computational effort required to iterate over the other independent variables, since there are no repeated computations with regard to those. Performing those computations sequentially would take hundreds of hours. To obtain results rapidly, the Phoenix computing cluster, which is part of the Partnership for an Advanced Computing Environment (PACE) at Georgia Tech, is employed. An HPC resource, such as the Phoenix cluster, offers the ability to utilize many dozens of computing cores in parallel. While each individual processor (i.e., core) might not perform any better than a consumer-grade processor, parallelization can provide tremendous speedup when solving large problems.

To optimally parallelize the problem, we apply Amdahl’s law, which predicts the theoretical speedup that is possible from using multiple processors [49]. With the HPC resources, it is possible to assign an arbitrary number of cores to solve each P-TPL FE model. If each simulation was fully parallelizable, the resulting speedup would scale linearly with the number of cores. However, because there is always some serial fraction *f* that cannot be parallelized, the speedup obtained by assigning *k* > 1 processors to each model is limited to 1/(*f* + (1 − *f*)/*k*) [50]. In the case of time-dependent FE simulations, there is a significant serial fraction that is impossible to parallelize. This results in a sublinear scaling of speedup with the number of processors. Thus, for solving the P-TPL models, it is more efficient to assign only a single processor to solve each model. This approach maximizes the total number of independent models that can be solved in parallel. It leads to an overall faster solution, even if each simulation takes slightly longer. Therefore, each width-power-[PI]_0_ combination is assigned a single, dedicated processor. On each processor, the dynamic programming approach is applied to build up the solutions for successive exposure values. Through the full exploitation of parallelization, 240 processors are used to parallelly generate results for the 240 width-power-[PI]_0_ combinations. Each processor requires approximately 90 min to complete its simulation tasks and it is possible to run as many of them in parallel as allowed by the HPC node availability.

## 3. Results and Discussion

### 3.1. Effect of Number of Pulses

**Figure 2:**
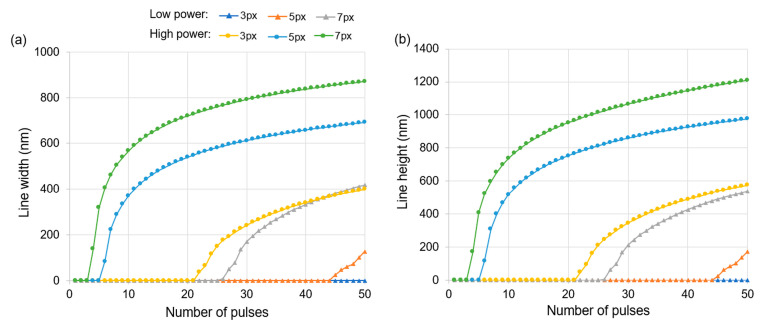
Size of the simulated central lines versus number of laser pulses for various projected linewidths (in px) and average optical power of the beam. (**a**) Line width versus number of pulses and (**b**) line height versus number of pulses. [PI]_0_ was held constant at 2.00 mol m^−3^. Low beam power was 47 nW/px and high beam power was 140 nW/px. The same plot legend applies to both plots.

The effect of the number of pulses (i.e., the exposure) on the width and height of the simulated lines is illustrated in Figure 2. At a lower power (i.e., 47 nW/px), longer exposures are required before any printing is observed with 5 px and 7 px lines, while 3 px lines do not generate prints at any exposure in the tested range. This observation is consistent with our past work, wherein it was demonstrated that thinner projected lines have a lower intensity [21]. Increasing the beam power (to 140 nW/px) results in rapid printing with low exposure. This observation is consistent with our expectation that higher optical dosages would lead to larger features. Interestingly, the accessible design space is reduced at a higher power, because small increases in exposure lead to large increases in feature size. For example, at the onset of printing with 7 px lines, an additional pulse increases the width by 180 nm at a higher power, but it increases the width by only 27 nm at a lower power. This effect is observed to scale with the width of the projected lines, and it is less prominent with thinner lines. Thus, printing with a small 3 px line at a higher power allows for sufficiently precise size control within the sub-100 nm regime for both width and height, while also being faster than printing with a large 7 px projection at a lower power. Overall, this dataset validates the approach of using a combination of tunable process parameters to increase the available design space for rapid sub-100 nm 3D printing.

### 3.2. Effect of Photoinitiator Concentration

**Figure 3:**
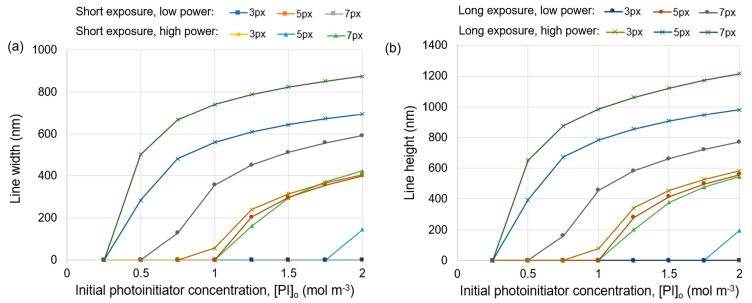
Size of the simulated central lines versus initial concentration of the photoinitiator ([PI]_0_) for various projected linewidths (in px), optical powers, and exposures. (**a**) Line width versus [PI]_0_ and (**b**) line height versus [PI]_0_. Short exposure was 10 pulses and long exposure was 50 pulses. Low power was 47 nW/px and high power was 140 nW/px. The same plot legend applies to both plots.

The effect of the initial photoinitiator concentration ([PI]_0_) on the simulated width and height of the features is shown in Figure 3. While short exposures and low beam powers do not result in printing with any [PI]_0_ value in the tested range, other combinations of exposure, power, and projected linewidth result in printing. The feature size increases when [PI]_0_ increases and this relationship is approximately logarithmic. This is similar to the logarithmic relationship between the feature size and the exposure time shown in Figure 2. The increase in feature size with an increasing [PI]_0_ is consistent with our expectation that more radicals will be generated in a resin that contains more photoinitiator, and that this will lead to more printing, even when the optical dosage is held constant. The logarithmic dependence is consistent with the exponentially varying intensity distribution in the projected image [21,35]. Notwithstanding the similarities in the qualitative effects of exposure and [PI]_0_ on the feature size, the two effects are fundamentally different. This is because [PI]_0_ is a continuous variable, whereas exposure is a discrete variable. In theory, the amount of photoinitiator in the resin can be tuned with arbitrary precision to achieve feature sizes between those obtained here through the use of intermediate concentrations. However, it is substantially harder to control or alter [PI]_0_ while printing, thereby limiting the practical use of this effect.

### 3.3. Effect of Optical Power

**Figure 4:**
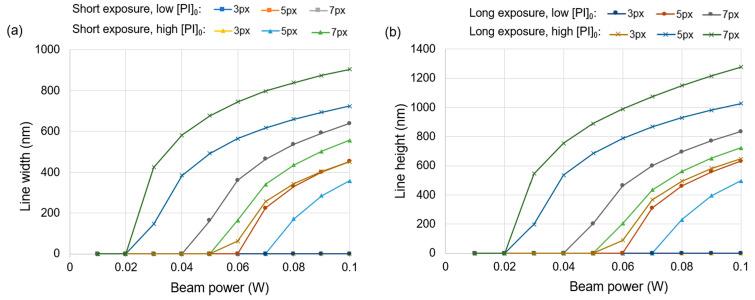
Size of the simulated central lines versus total optical power of the beam for various projected linewidths (in px), [PI]_0_, and exposures. (**a**) Line width versus optical power and (**b**) line height versus optical power. Low [PI]_0_ was 0.50 mol m^−3^ and high [PI]_0_ was 2.00 mol m^−3^. Short exposure was 10 pulses and long exposure was 50 pulses. 1 W of total optical power of the beam was equivalent to 1560 nW/px of optical power in the simulated P-TPL system. The same plot legend applies to both plots.

The effect of the optical power of the beam on the simulated width and height of the printed features is shown in Figure 4 for four different exposure–[PI]_0_ combinations. With a short exposure and low [PI]_0_, printing cannot be obtained at any beam power or linewidth. It is observed that printing with 5 px and 7 px projections is feasible at higher beam powers when combined with a higher [PI]_0_ or longer exposure. Printing is not feasible with 3 px lines unless a high [PI]_0_ and long exposure are used simultaneously. Overall, these trends are consistent with the expectation that more printing will occur at a higher optical power. Although the qualitative effects of power are similar to the effects of exposure and [PI]_0_, size control via power is more advantageous. Not only is it possible to continuously adjust the power, but it is also much easier to adjust than [PI]_0_. Depending on practical issues associated with the specific printing setup, there could be some margin of error in the extent to which tuning the feature size is possible, possibly stemming from the ability of the system to keep the other conditions constant while adjusting the power. It stands to reason that tuning along a feature size versus power curve with a gentle gradient would allow for easier feature size control, since small changes in power would not result in large changes in feature size. Thus, Figure 4 is also useful in selecting the best exposure–[PI]_0_ combination for printing in a particular feature size range, depending on how steep the corresponding power curve is at that point.

### 3.4. Sub-100 nm Regime

**Figure 5:**
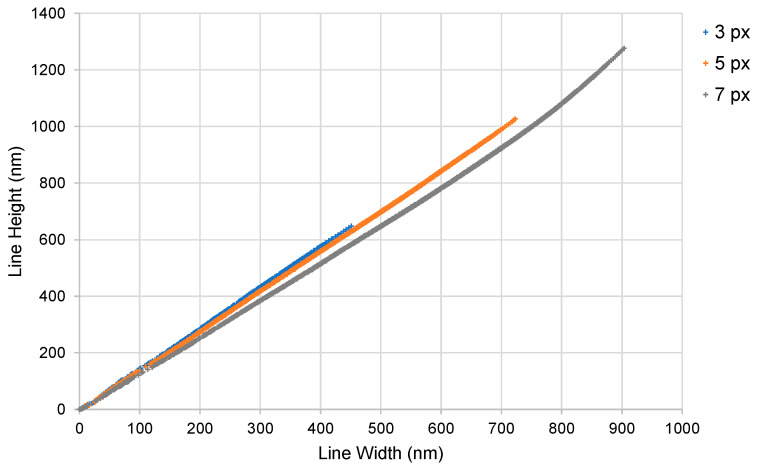
Simulated height versus width of the central lines for all 11,760 combinations of parameters that were studied here.

If the hypothesis that it is possible to achieve arbitrary feature sizes through the interpolation of power or [PI]_0_ is correct, then it is expected that the 11,760 parameter combinations, taken together, should yield a continuous feature size distribution. The data shown in Figure 5 demonstrate that this is indeed the case. Plotting all 11,760 datapoints shows that having multiple independent variables to modify allows for printing of virtually any feature size, including in the sub-100 nm regime. The presence of datapoints right down to <10 nm suggests that there is no physics-based minimum feature size limit for P-TPL. The limit of a printing system likely arises only from the practical difficulties associated with printing and developing structures of nanometer scales. However, the density of points in this sampling is lower in the sub-100 nm regime and it arises from the logarithmic curves seen in Figure 2, Figure 3 and Figure 4. This suggests that, while printing in this regime is possible, it would require the careful calibration and control of printing conditions to achieve a precise sub-100 nm print.

The banded distribution of feature sizes corresponds to the slightly different aspect ratios of the 3 px, 5 px, and 7 px lines. This aspect ratio is the ratio of the height and width of each simulated line feature. A change in the aspect ratio is observed for the larger lines, which is most notable in the 7 px dataset. The slight decrease in the aspect ratio of the 3 px versus 7 px lines is consistent with the corresponding decrease in the aspect ratio of the optical intensity distribution [21]. In the sub-200 nm regime, the simulated aspect ratio varies between 1.3 and 1.4. This value matches closely with past experimental data, wherein an aspect ratio of 1.3 was observed for features below 200 nm in size [21]. In past experiments, it was observed that the aspect ratio can increase to as much as 4.3 for lines wider than 500 nm [21]. Our simulations are unable to capture this increase in the aspect ratio for the wider lines. We suspect that this discrepancy arises because the larger cured features can start absorbing some additional light in the single-photon mode, which is absent in the uncured resin. Additional work is necessary to clarify this issue. Nevertheless, our model can accurately capture the effects in the sub-200 nm regime; therefore, it can effectively characterize the minimum feature size limits of P-TPL.

**Figure 6:**
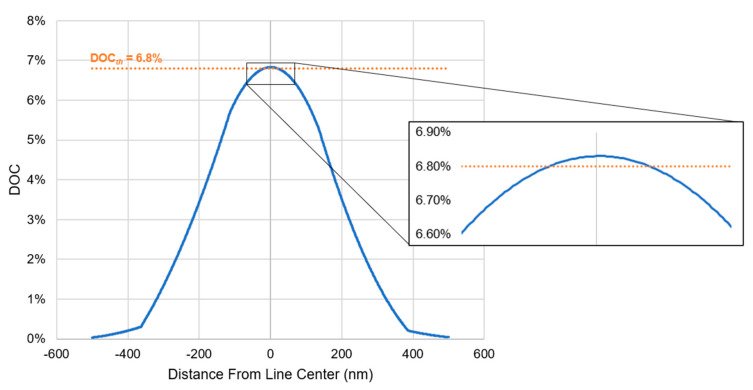
*DOC* versus distance from the center of the central line along the width direction for one of the parameter combinations which led to a simulated linewidth of 21 nm.

Our simulation demonstrates that, based on the photopolymerization mechanism, a theoretical limit of the minimum feature size does not exist for P-TPL. However, the sensitivity of the printing outcome to the processing conditions will lead to a practical limit. The origin of this practical limit can be better appreciated by analyzing the *DOC* profile of the line features. The variation in the *DOC*, along the width of the central line for one of the 11,760 simulated parameter combinations, is shown in Figure 6. When an optical dosage is delivered to the system, the *DOC* curve is pushed upwards. If any region of the curve crosses the *DOC_th_*, printing is observed in that region. This particular line has a peak *DOC* just above the threshold *DOC_th_*, giving a predicted linewidth of only 21 nm. The *DOC* versus distance curve has an exponential profile, which arises from the exponential intensity distribution. With such a profile of the *DOC*, the spatial gradient of the *DOC* is zero at the center, and it increases as the distance from the center of the line increases. For thin lines, the boundary of the line lies close to the center, where the *DOC* gradient is weak. In contrast, the boundary of a thicker line lies farther away from the center, where the *DOC* gradient is strong. This means that an infinitesimal increase in the *DOC* will lead to a larger increase in the width for the thinner feature versus the thicker feature. Therefore, the sensitivity of printing to the processing conditions increases as the width of the lines decreases. Thus, to achieve fine printing in practice, one must either increase the curvature (i.e., equivalent to the gradient of gradient) of the *DOC* profile or finely and precisely control the step increases in the *DOC*.

### 3.5. Insights for Rapid Sub-100 nm 3D Printing

If rapid 3D printing is desired in the sub-100 nm regime, one must perform printing with low exposures, i.e., with a low number of pulses. However, by doing so, one would encounter a tradeoff with the degree of size tunability. This tradeoff arises because each pulse must deliver a high dosage to compensate for the low number of pulses. As each pulse would carry a higher dosage, the feature size would increase by larger steps with an increasing number of pulses, provided that all the other parameters are held constant. This tradeoff can be overcome by recognizing that the dosage can be independently tuned by changing the projected linewidth, the optical power, and the photoinitiator concentration. The power and linewidth control the optical dosage, whereas the photoinitiator concentration controls the chemical dosage. By simultaneously varying these three parameters and applying short exposures, one can finely tune the linewidth in the sub-100 nm regime. If large projected linewidths are chosen, then moderate levels of power and photoinitiator concentration would be required to achieve the desired dosages. In contrast, if small projected linewidths are chosen, then high levels of power and photoinitiator concentration would be required. Out of these two choices, the conditions with larger linewidths would be less sensitive to the process parameters due to the higher curvature of the intensity distribution. This higher curvature can be verified from the optical simulations in our past work [21]. Therefore, wider projections are expected to be more stable to experimental noise, and are more likely to be practically printable. Thus, our simulations presented here can guide the rational selection of process parameters to achieve rapid sub-100 nm 3D printing.

### 3.6. Demonstration of Explanatory Power of the Simulations

As our simulations were performed over a vast parameter space, the results presented here can explain seemingly counterintuitive empirical observations during the printing of fine features with P-TPL. To demonstrate the explanatory power of our simulations, we printed a set of widely separated line features under various processing conditions. Specifically, we varied the projected linewidth and duration of exposure (i.e., the number of pulses) while holding the optical power and photoresist composition as fixed. Thus, the design of our experiments matched the design of the simulations summarized in Figure 2. The projected lines were either 3 px, 5 px, or 7 px wide. For each linewidth, the number of pulses was varied from 1 pulse to 14 pulses in steps of 1 pulse. The nominal optical power was 157 nW/px, and this represented the average power of the beam when averaged over the entire area of projection. The photoinitiator was a custom molecule 4,4′-((1E,1′E)-(2-((2-Ethylhexyl)oxy)-5-methoxy-1,4-phenylene)bis(ethene-2,1-diyl))bis(N,N dibutylaniline), and its initial concentration in the photoresist was 1.65 mol m^−3^. The relevant properties of this photoinitiator are listed in Table 1. The monomer blend of the photoresist comprised a mixture of multifunctional acrylates that were mixed in proportion to achieve a refractive index of 1.52. Specifically, the monomer comprised a mixture of pentaerythritol tetraacrylate, pentaerythritol triacrylate, trimethylolpropane triacrylate, and bisphenol A ethoxylate diacrylate with an average Mn ∼468—EO/phenol 1.5. Further details of the photoresist composition are available elsewhere [35].

**Figure 7:**
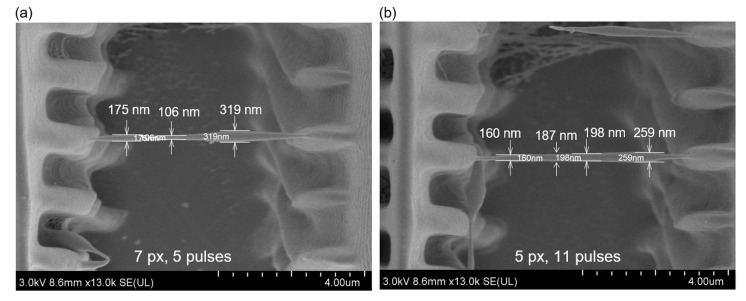
Scanning electron microscope images of physically printed nanowires. (**a**) Printed with 7 px wide line projections and with an exposure of 5 pulses and (**b**) printed with 5 px wide line projections and with an exposure of 11 pulses. For both prints, the nominal optical power was 157 nW/px and the [PI]_0_ was 1.65 mol m^−3^.

As the numerical values of the process parameters in our experiments were different from those in our simulations, we did not expect our experimental observations to quantitatively match the simulation predictions. Nevertheless, our simulations could qualitatively predict the process outcome, despite the differences in the processing conditions. For example, the simulations summarized in Figure 2 predicted that the threshold exposure would increase with an increase in the projected linewidth. This prediction was validated by our experiments, wherein we observed that the threshold exposure was 5 pulses for 7 px wide projections and 11 pulses for 5 px wide projections. The 3 px wide projections did not generate any nanowires, even at an exposure of 14 pulses. Scanning electron microscope (SEM) images of the nanowires that were printed at the threshold exposure, corresponding to the 7 px and 5 px wide projections, are shown in Figure 7. These observations validate the predicted scaling of the threshold exposure with the projected linewidth and demonstrate the explanatory power of the simulations.

The explanatory power of the simulations can be further appreciated by applying the results of the simulations to understand the variations in the linewidth that were observed in the experiments. The nanowires shown in Figure 7 varied in width along their length due to variations in the intensity of the beam at the different locations within the projected line. These variations arose due to the practical challenges that were involved in generating a perfectly flat beam profile. From the SEM images shown in Figure 7, one can deduce that the pixels located at the periphery of the nanowires had a higher power per pixel when compared to the pixels at the center, and that the pixels on the right had the highest power. This scaling of power can be deduced from the scaling of the printed linewidths. What is interesting in these experiments is that, at the low-power central locations, the width of the nanowire printed with 7 px wide projections was smaller than the width of the nanowire printed with 5 px wide projections (i.e., 106 nm versus 187 nm). However, the width of the 7 px wide nanowire was larger than the 5 px wide nanowire in the high-power right-side peripheral locations (319 nm vs 259 nm). Thus, a finer nanowire segment could be printed with the 7 px projections, but the variation in linewidth was also higher with the 7 px projections. This seemingly self-contradictory empirical observation can be satisfactorily explained by the simulations presented here. From Figure 2, one can observe that, for the low-power condition, the simulated width of the 7 px nanowire at the threshold exposure was smaller than the width of the 5 px nanowire at the threshold (i.e., 7 nm at 24 pulses versus 25 nm at 45 pulses). However, this trend reversed in the high-power condition, wherein the 7 px projections generated wider simulated nanowires than the 5 px projections at all exposures. This behavior arose from the discrete nature of the illumination in P-TPL, and it is scientifically feasible, because the optical dosage per pulse is lower in the low-power condition so that finer control of the feature size can be achieved by varying the number of pulses. Consequently, it is possible to achieve fine printing under these conditions, but the feature size becomes highly sensitive to the variations in the optical power. Thus, by effectively explaining the empirical observations, the simulations presented here can help to guide P-TPL process optimization to rapidly print fine features with high precision.

## 4. Conclusions

Here, we presented a dynamic programming scheme to rapidly simulate photopolymerization in P-TPL for more than 10,000 combinations of process parameters. We investigated the effects of projected linewidth, beam power, photoinitiator concentration, and exposure on the width and height of the lines. When only one parameter was varied at a time, there existed a tradeoff between the rate of printing and the degree of size tunability. This tradeoff could be overcome by simultaneously varying multiple process parameters. Thus, despite the discrete nature of the pulsed projection, we observed that there is no physics-based limit on the minimum feature sizes attainable with P-TPL, even at high rates of printing. Nonetheless, the strong thresholding effect seen in TPL means that it is necessary to precisely control the optical dosage to achieve the desired feature size, particularly for sub-100 nm prints. For rapid printing in the sub-100 nm regime, printing must be performed at low exposures with large projected linewidths and moderate levels of power and photoinitiator concentration. The predicted feature size curves presented here can help in choosing an optimal parameter combination for printing in various desired feature size regimes. Thus, our work can significantly reduce the iterative guesswork in the P-TPL-based 3D printing of functional micro- and nano-scale structures.

## Figures and Tables

**Table 1:** Model parameters for this study.

Symbol	Parameter Name	Value	Source
*σ* ^(2)^	Two-photon cross section of photoinitiator	133 × 10^–50^ cm^4^s/photon-molecule	Estimate from Rumi et al. (Figure 5, compound **8**) [44]
*h*	Planck’s constant	6.626 × 10^–34^ m^2^ kg s^−1^	Fundamental constant
*k_p_*	Polymerization rate constant	4.3 × 10^4^ dm^3^ mol^–1^ s^–1^	Mueller et al. [32]
*k_q_*	R* quenching rate constant	2.3 × 10^6^ dm^3^ mol^–1^ s^–1^	Mueller et al. [32]
*k_t_*	Termination rate constant	5.9 × 10^4^ dm^3^ mol^–1^ s^–1^	Calibrated from experiments [35]
Φ	Quantum yield of photoinitiator	0.0061	Calibrated from experiments [35]
*DOC_th_*	Degree of conversion threshold	0.068	Directly measured [35]
*D*_O2_	Diffusivity of oxygen	1.2 × 10^−12^ m^2^ s^–1^	Estimated using Stokes–Einstein (S-E) equation
*D*_R*_	Diffusivity of R*	10^−13^ m^2^ s^–1^	Estimated using S–E equation
*v*	Optical frequency (central)	375 THz	Determined by laser in the printer

**Table 2:** List of process parameters that were varied in this study.

Parameter	List of Values
Width of projected lines (px)	[3, 5, 7]
Exposure (number of pulses)	[2, 3, … 49, 50]
Average optical power of the beam (μW per px)	[0.01, 0.02, … 0.09, 0.10] × 1.56
Photoinitiator concentration (mol m^−3^)	[0.25, 0.50, … 1.75, 2.00]

## Data Availability

The data presented in this study are available on request from the corresponding author.
